# High Intensity Training to Treat Chronic Nonspecific Low Back Pain: Effectiveness of Various Exercise Modes

**DOI:** 10.3390/jcm9082401

**Published:** 2020-07-27

**Authors:** Jonas Verbrugghe, Anouk Agten, Sjoerd Stevens, Dominique Hansen, Christophe Demoulin, Bert O. Eijnde, Frank Vandenabeele, Annick Timmermans

**Affiliations:** 1 REVAL—Rehabilitation Research Center, BIOMED, Faculty of Rehabilitation Sciences, Hasselt University, 3500 Hasselt, Belgium; anouk.agten@uhasselt.be (A.A.); sjoerd.stevens@uhasselt.be (S.S.); dominique.hansen@uhasselt.be (D.H.); bert.opteijnde@uhasselt.be (B.O.E.); frank.vandenabeele@uhasselt.be (F.V.); Annick.Timmermans@uhasselt.be (A.T.); 2 Salvatorstraat 20, 3500 Hasselt, Jessa Hospital, 3500 Hasselt, Belgium; 3 Department of Sport and Rehabilitation Sciences, University of Liege, 4000 Liege, Belgium; christophe.demoulin@uliege.be

**Keywords:** chronic low back pain, exercise therapy, high-intensity training, clinical trial

## Abstract

High-intensity training (HIT) improves rehabilitation outcomes such as functional disability and physical performance in several chronic disorders. Promising results were also found in chronic nonspecific low back pain (CNSLBP). However, the impact of different exercise modes on HIT effectiveness in CNSLBP remains unclear. Therefore, this study evaluated the effectiveness of various HIT exercise modes and compared differences between these modes, on pain intensity, disability, and physical performance, as a therapeutic intervention for persons with CNSLBP. In a randomized comparative trial, consisting of a 12-week program, persons with CNSLBP were divided into four HIT groups, i.e., cardiorespiratory interval training coupled with either general resistance training, core strength training, combined general resistance and core strength training, or mobility exercises. Before and after the program, the Numeric Pain Rating Scale (NPRS), Modified Oswestry Disability Index (MODI), and Patient Specific Functioning Scale (PSFS) were recorded, and a cardiopulmonary exercise test (VO_2_max, cycling time) and isometric trunk strength test (maximum muscle torque) were performed. Eighty participants (mean age: 44.0 y, 34 males) were included. Improvements were found within all groups after the HIT programs and ranged from −39 to −57% on the NPRS, +27 to +64% on the MODI, +38 to +89% on the PSFS, +7 to +14% on VO_2_max, and +11 to +18% on cycling time. No differences between groups were found. High-intensity cardiorespiratory interval training improves CNSLBP rehabilitation outcomes when performed with other HIT exercise modes or mobility exercises. Hence, when setting up an exercise therapy program in CNSLBP rehabilitation, various HIT modes can be considered as therapy modalities.

## 1. Introduction

Chronic low back pain is one of the most common musculoskeletal disorders worldwide [1,2,3,4], with a point prevalence of around 20% [5]. It is currently ranked as the number-one cause of long-term disability, leads to more than 10% of all work absenteeism, and is a considerate financial burden for healthcare systems [6,7,8,9]. Up to 85% of chronic low back pain cases are nonspecific, with no clear underlying pathoanatomical cause. The management of chronic nonspecific low back pain (CNSLBP) consists of a multidimensional treatment approach that includes exercise therapy as an important part [10,11]. Nevertheless, studies comparing various exercise therapy protocols in CNSLBP have not been able to unequivocally show which exercise mode is most effective for decreasing primary outcomes such as pain and disability [10,12,13,14]. As a result, clinical guidelines lack instructions or give conflicting recommendations toward the preferred exercise mode in this population [11,15]. Furthermore, overall effect sizes of exercise therapy in CNSLBP treatment remain only small to moderate [16].

One of the reoccurring hypotheses for these substandard effect sizes of exercise therapy in CNSLBP is that suboptimal training intensity attenuates the therapy outcomes [15,17]. Interestingly, high-intensity training (HIT) has already been shown to improve physical outcome measures, such as exercise capacity and muscle strength, whilst also decreasing health-related parameters and disorder-related disability more than equal training modes performed at lower intensities in a whole spectrum of persons with chronic disorders, such as axial spondyloarthritis [18], multiple sclerosis [19], heart failure [20], Chronic Obstructive Pulmonary Disease [21], and cardio-metabolic diseases [22]. In like manner, a systematic review by Wewege et al. recently indicated that increased effectiveness of exercise therapy might also be expected in CNSLBP rehabilitation when higher training intensities are used [14].

When employing HIT in rehabilitation, either a cardiorespiratory protocol or a strength protocol is mostly chosen, based on the specific needs of the disorder. High-intensity cardiorespiratory interval training can improve maximal oxygen intake [23], and it could thereby decrease physical deconditioning that might be seen in CNSLBP [24]. High-intensity general resistance and core strength training can improve muscle strength and thus decrease deconditioning of the trunk and extremity muscles that has been reported in CNSLBP [25,26]. Thereby, both these modes could provide improvements on functional abilities. In healthy persons and other patient populations, combined HIT programs have shown even more value [27]. However, currently, there is limited knowledge on differences between training modes and the effect of combining them in CNSLBP [14].

In this respect, Verbrugghe et al. already evaluated the effectiveness of a combined high-intensity protocol of cardiorespiratory interval, general resistance, and core strength training in CNSLBP rehabilitation and found greater improvements on disability and exercise capacity, compared to a similar protocol performed at moderate intensity [28]. Hence, the increased physical stimulus of HIT clearly provided an added effectiveness in CNSLBP. However, while this study demonstrated the benefits of using higher intensity during a combined exercise therapy protocol, it did not provide the ability to distinguish the value of various separate exercise modes of HIT. Consequently, it remains unclear whether there are differences in therapy effectiveness when performing different HIT protocols in CNSLBP and whether combined protocols provide added value compared to more isolated protocols in this population. Therefore, the objectives of the current study were to (1) evaluate the effectiveness of four therapy modes of HIT on pain intensity, disability, exercise capacity, and isometric abdominal/back muscle strength and (2) compare the differences in effectiveness between these modes as a therapeutic intervention for persons with CNSLBP.

## 2. Materials and methods

### 2.1. Trial Design

**Figure 1:**
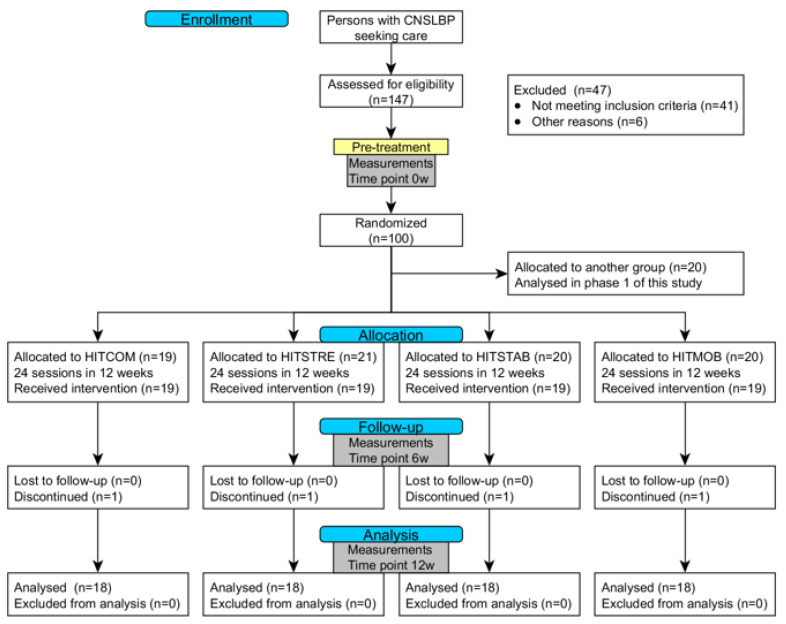
CONSORT flowchart of the research design. Abbreviations: CNSLBP = chronic nonspecific low back pain; HITCOM = high-intensity cardiorespiratory training combined with high-intensity general resistance and high-intensity core strength training; HITSTRE = high-intensity cardiorespiratory training combined with high-intensity general resistance training; HITSTAB = high-intensity cardiorespiratory training combined with high-intensity core strength training; HITMOB = high-intensity cardiorespiratory training combined with trunk mobility exercises.

The present randomized comparative trial is part of a larger project entitled *“*High intensity training in chronic nonspecific low back pain”, which evaluates the effects of training intensity and various training modes in CNSLBP rehabilitation through a prospectively registered, five-arm, RCT organized at REVAL (Hasselt University, Diepenbeek, Belgium). The results of the effectiveness of HIT in comparison to a moderate-intensity training group have been published previously [28]. The current manuscript describes phase two, which evaluates the difference between four exercise modes of HIT. A comprehensive research design flowchart is displayed in Figure 1. This project was approved by the Medical Ethics Committee of Jessa Hospital (Hasselt, Belgium) and registered at clinicaltrials.gov as NCT02911987.

### 2.2. Participants and Recruitment

Persons with CNSLBP in the area of Limburg (Belgium) were recruited through local advertisements. To be eligible, persons had to speak Dutch, be 25–60 years old, and have CNSLBP (i.e., pain localized below the costal margin and above the inferior gluteal folds with or without referred leg pain of a nociceptive mechanical nature, not attributable to a recognizable, known specific spinal pathology, for a period of at least twelve weeks [3,29]). Persons were excluded when they had a history of spinal fusion, had a musculoskeletal disorder aside from CNSLBP that could affect the correct execution of the therapy program, had co-morbidities (e.g., paresis and/or sensory disturbances by neurological causes), were pregnant, had ongoing compensation claims and/or a work disability >six months, had followed an exercise therapy program for low back pain in the past three months, or were not able to attend regular therapy appointments. Interested persons received a patient information letter and were invited for an intake session by one of the researchers. During the intake session, the information letter was reviewed, study inclusion and exclusion criteria were evaluated, the informed consent was signed, and a study-specific screening form concerning red flags for low-back-pain rehabilitation was filled out.

### 2.3. Randomization and Blinding

Participants were randomly assigned to one of four experimental groups performing high-intensity cardiorespiratory interval training coupled with either high-intensity general resistance training (HITSTRE), high-intensity core strength training (HITSTAB), a combined high-intensity general resistance and core strength program (HITCOM), or mobility exercises (HITMOB). A permuted block randomization with a block size of four was used to foresee equal group division (1:1 allocation ratio). To ensure concealment of allocation, a research assistant not involved in the study picked a sealed opaque envelope containing the allocated group for each participant. Given the nature of the exercise therapy, it was not possible to blind participants and physiotherapists for group assignment. To limit performance bias of the participants, the study was described to the participants as “a comparison between different modes of exercise therapy treatments”, and participants were informed that equal progression was expected in each group.

### 2.4. Interventions

Participants of all groups were enrolled in a 12-week exercise therapy program consisting of 24 supervised individual therapy sessions (2 × 1, 5 h/week). The training volume of each group was equal. A manual with protocols, exercises, and progression definitions was provided to the physiotherapists to assure standardized follow-up of all participants. Sessions missed by the participants because of adverse events were registered.

Each group followed the same cardiorespiratory training consisting of an interval protocol on a cycle ergometer. After a five-minute warm-up, interval training started, consisting of five one-minute bouts (110 repetitions per minute at 100% VO_2_max workload), separated by one minute of active rest (75 RPM at 50% VO_2_max workload). Cycling bouts increased every two sessions by 10′′ up to 1′50′′ after 12 sessions. Recovery time (1′) between bouts remained stable. This protocol was repeated from session 13 to 24 with an updated workload, based on the results from a complementary cardiopulmonary exercise test. Apart from the cardiorespiratory training, each group performed a specific additional therapy protocol. These additional protocols are explained below.

**Figure 2:**
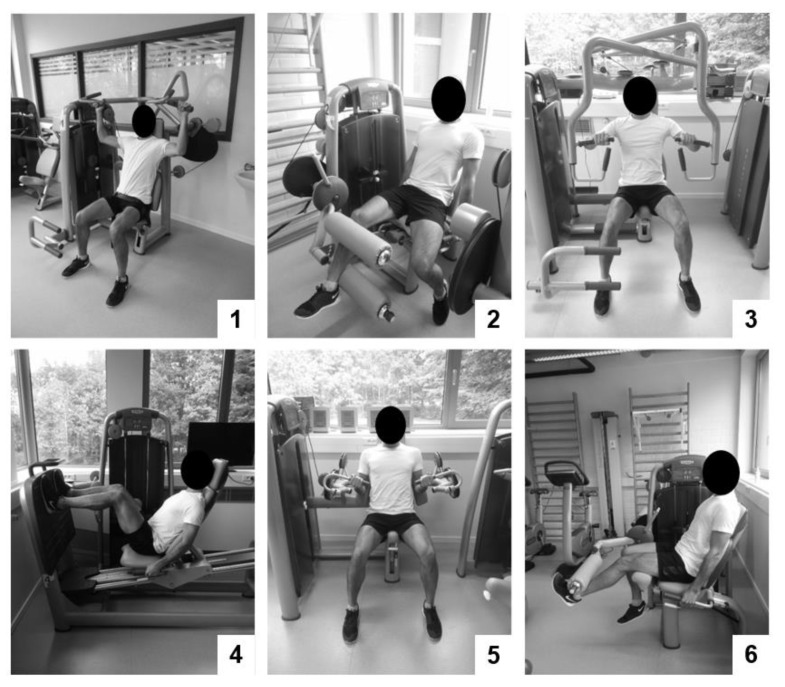
Strength exercises in the limb strength protocol: (**1**) vertical traction, (**2**) leg curl, (**3**) chest press, (**4**) leg press, (**5**) arm curl, and (**6**) leg extension.

**Figure 3:**
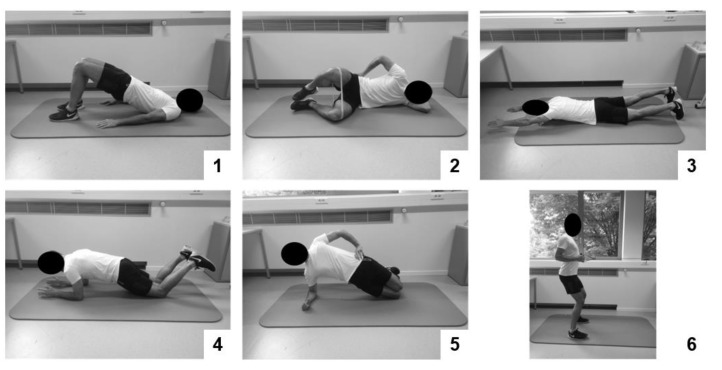
Baseline strength exercises in the core strength protocol: (**1**) glute bridge, (**2**) glute clam, (**3**) lying diagonal back extension, (**4**) adapted knee plank, (**5**) adapted knee side plank, and (**6**) shoulder retraction with hip hinge.

*Therapy group 1 performed high-intensity general resistance training and high-intensity core strength training (HITCOM).* General resistance training consisted of three upper- and three lower-body exercises executed on fitness devices (Figure 2). During the first session, exercises were explained and demonstrated by the physiotherapist. Then, exercises were repeated by the participant, while movement corrections were made by the physiotherapist. On the second training session, a one repetition maximum (1RM) testing was performed for each exercise. During the following sessions, one set of a maximum of twelve repetitions was performed at 80%1RM for each exercise. Researchers progressively increased the exercise weight when the participant was able to perform more than 10 repetitions on two consecutive training sessions. Core strength training consisted of six static core exercises (Figure 3). Exercises were chosen in function of their ability to load the core muscles at an intensity of at least 40–60% of the maximum voluntary contraction [30]. On the first session, patients were educated on isolated activation of specific core muscles (m. transversus abdominis, m. multifidus, and m. gluteus). On the second session, the physiotherapist explained and demonstrated the exercises. The participant then repeated the exercises while movement corrections were made. During the following sessions, participants performed one set of ten repetitions of a ten-second static hold. Participants were encouraged to hold the last repetition for as long as possible. Exercises were made more difficult by increasing the static hold time and progressing to a more demanding posture when they were executed with a stable core posture for the indicated time by the participant on two consecutive training sessions.

*Therapy group 2 performed high-intensity general resistance training (HITSTRE).* General resistance training was identical to the general resistance training protocol described in HITCOM; however, each circuit of exercises was executed twice, starting from session three, throughout the program.

*Therapy group 3 performed high-intensity core strength training (HITSTAB).* Core strength training was identical to the core strength training protocol described in HITCOM; however, each circuit of exercises was executed twice, starting from session three, throughout the program.

*Therapy group 4 performed trunk mobility exercises (HITMOB).* Trunk mobility exercises consisted of six exercises aimed to improve the mobility of the trunk and hip complex in general (hamstrings stretch, gluteus medius stretch, lower back rotation mobilization, back extension stretch, hip flexor stretch, and mid-back extension mobilization). Stretches were held on each side twice for 30 s. Mobilizations were performed twice for ten repetitions.

### 2.5. Testing Procedure and Outcomes

The following demographic and clinical characteristics were collected at baseline: gender, age (years), weight (kg), and height (cm), to calculate BMI and time of onset of CNSLBP, fear of movement (by means of the 17-item Tampa Scale for Kinesiophobia (TSK)) and physical activity (by means of the Physical Activity Scale for Individuals with Physical Disabilities (PASIPD)) [31,32]. Outcomes were collected at baseline (PRE) and at the end of the training program (POST). An interim evaluation (MID) was executed after 12 sessions (these data were used only to perform an intention-to-treat analysis). The primary outcomes were disability and pain intensity, whereas the secondary outcome measures were function, exercise capacity, and muscle strength. All of these outcomes are considered to be reliable and valid in patients with CNSLBP [33,34,35].

The Modified Oswestry Disability Index (MODI) evaluated disability and consists of ten items scored on a five-point scale. The total score is expressed in percentage of disability (higher is more disability) and displays a degree of functional limitation.

The Numeric Pain Rating Score (NPRS) was used to evaluate average pain intensity in the previous six-week period by choosing a number of the 0–10 scale (zero means no pain, and ten means worst pain imaginable).

The Patient Specific Functioning Scale (PSFS) evaluated individual-specific functioning. Participants wrote down the three-to-five most relevant activities compromised because of a physical disability. These activities are rated on a 0–10 numeric rating scale (zero means unable to perform, and ten means able to perform at preinjury level). A mean percentage for all activities is calculated.

A maximal cardiopulmonary exercise test (75RPM) on an electronically braked cycle ergometer (eBike Basic, General Electric GmbH) was used to evaluate exercise capacity through maximal oxygen uptake (VO_2_max) and maximal workload through cycling time (min). Participants started at a low workload that gradually increased each minute (♂: 30 W + 15 W/min; ♀: 20 W + 10 W/min). Supplementary, respiratory exchange ratio (RER), and heart rate were determined through breath-by-breath gas-exchange analysis (Cortex MetaMax 3B) and heart-rate monitoring (Polar).

A maximal isometric muscle strength test of the trunk flexors and extensors, using an isokinetic dynamometer (System 3, Biodex, Enraf-Nonius [36]), was also included. Peak torque of trunk flexors and extensors was recorded during three maximal isometric trunk flexions and trunk extensions, respectively [37]. Peak torque was expressed in Newton meter (Nm) and normalized to bodyweight (Nm/kg).

### 2.6. Data Analysis

JMP Pro (12.0, SAS Institute Inc., Cary, USA) was used for data analysis. A sample size calculation was performed (80% power, alpha = 0.05) based on the observed within-group therapy effect of two points (SD = 2.5) on the NPRS in a previously published feasibility study [38], in which a similar HIT exercise therapy protocol was used. Given the calculated estimates, *n* = 15 was needed in each group to provide sufficient power to evaluate within group differences. As a 20% loss to follow-up was expected, a total amount of *n* = 72 participants (*n* = 18 in each group) was needed. Descriptive statistics were used to display baseline group characteristics. Normality and homoscedasticity of each outcome were checked by fitting a general linear model of the PRE–POST delta values and plotting the residuals to look for equal variance and symmetry, and identify possible outliers. Linear mixed models were fitted for each outcome measure, with time and group as covariates and incorporated random intercepts for the participants. Multiple comparisons were executed to evaluate group (baseline differences), time (within group differences), and interaction effects (between group differences). For all tests of significance, an α-level = 0.05 was used. For dropouts, an intention-to-treat analysis (ITT) was followed, using a last-observation-carried-forward approach (LOCF) [39], only if a MID measurement was performed. If no MID measurement was performed (dropout before 12 sessions of therapy), the participant was seen as missing data and was not used for further analysis. To check for selective dropout, differences between participants completing the trial and dropouts were examined (independent *t*-tests, Mann–Whitney U tests, and X^2^ tests).

## 3. Results

### 3.1. Recruitment and Baseline Data

A total of 147 persons seeking care for chronic low back pain were screened for eligibility. Of these, 104 persons entered the trial, while 43 persons did not meet the inclusion criteria. Four persons were not able to comply to the therapy program, due to conflicting job schedules or commuting problems, and were excluded before the start. Finally, 80 participants were randomized into the four HIT training groups: HITCOM: *n* = 19; HITSTRE: *n* = 21; HITSTAB: *n* = 20; and HITMOB: *n* = 20. In general, more women (60%) were included than men. Mean age was 44.1 years (SD =9.7), and pain onset was 13.4 years (SD = 9.1). Study groups were well matched at baseline, and no group effects were found (*p* > 0.05), except for symptom duration due to a lower symptom duration in HITSTAB (*p* = 0.001). However, all groups clearly included patients with a long-lasting chronic disorder (range: 8.8–15.8 years). Nonetheless, all treatment effects were adjusted for baseline estimates. An overview of patient characteristics at baseline is displayed in Table 1.

### 3.2. Treatment Adherence, Dropouts, and Adverse Events

Mean session attendance of 23.4 (SD = 1.07) out of a possible 24 sessions. No differences in treatment adherence were found between the groups. Six dropouts (8% of all participants) were noted. One person did not start the therapy sessions after the baseline assessment, due to practical issues. Five persons dropped out during the therapy protocols, of which three did so due to long-term sickness not related to low back pain, and two due to practical issues concerning the execution of two weekly training sessions. None of the dropouts performed a MID measurement needed for an ITT. No differences in baseline characteristics were found between the dropouts and other participants. Participants did not report any adverse events which could have affected the correct fulfillment of this study.

### 3.3. Outcomes after the Training Program

An overview of the results is presented in Table 2.

Disability measured by the MODI improved within all groups (*p* < 0.001). Improvements ranged from a 5.4-point reduction (27% difference) in HITSTRE to a 14.6-point reduction (57% difference) in HITCOM. No between-groups differences (*p* = 0.107) were found.

Pain intensity measured by the NPRS improved within all groups (*p* < 0.001). Improvements ranged from a 2.0-point reduction (39% difference) in HITSTRE to a 3.4-point reduction (89% difference) in HITMOB. No between groups differences (*p* = 0.176) were found.

Functioning measured by the PSFS improved within all groups (*p* < 0.001). Improvements ranged from a 18% reduction (33% difference) in HITSTAB to a 32% reduction (83% difference) in HITMOB. No between-groups differences (*p* = 0.334) were found.

Exercise capacity measured by VO_2_max and cycling time. VO_2_max improved within all groups (*p* < 0.001). Improvements ranged from a 2.4 mL/kg/min (7% difference) in HITSTAB to a 4.9 mL/kg/min (14% relative difference) in HITMOB. Cycling time improved within all groups (*p* < 0.001). Improvements ranged from a 1.6 min increase (11% difference) in HITSTRE to a 2.5 min increase (18% difference) in HITCOM. No between groups differences were found in either VO_2_max (*p* = 0.191) or cycling time (*p* = 0.193).

Regarding muscle strength, both abdominal and back strength were evaluated. Abdominal strength only improved within group () in HITMOB (0.17 Nm/kg; 12% difference). Back strength only improved within group () in HITCOM (0.31 Nm/kg; 10% difference). No differences between groups were found in either abdominal (*p* = 0.218) or back strength (*p* = 0.308).

## 4. Discussion

This study was the first clinical trial to evaluate the effectiveness of various HIT exercise modes and compare the differences in effectiveness between these modes as a therapeutic intervention for persons with CNSLBP. Equal improvements were noted in all groups on disability, pain intensity, function, and exercise capacity. Therapy effect sizes were statistically significant within each group. Furthermore, clinically relevant within-group differences were found in each group, for the primary outcomes (i.e., more than 30% for disability and pain [40]) and some secondary outcomes (i.e., more than 20% for functioning [41] and more than 3,5 mL/kg/min for VO2max [42]). However, no minimal clinically relevant differences or even statistically significant differences were found between the groups. As such, the authors argue that differences between groups were negligible. Results of this study show the promising value of HIT in CNSLBP rehabilitation, whilst they also reveal the lack of difference between various exercise modes of HIT in this population. Based on these results, cardiorespiratory high-intensity interval exercise might be performed with or without other frequently used exercise therapy modes, potentially according to personal factors, such as patients’ preferences or environmental factors, such as therapist experience or equipment availability.

Differences in effectiveness of specific exercise therapy modes in CNSLBP rehabilitation were already examined in several systematic reviews, but results have been inconsistent. For instance, Searle et al. showed that general resistance and core strength training had a beneficial effect over other exercise therapy programs on improving pain and decreasing disability [15]. Furthermore, this study stated cardiorespiratory and combined exercise therapy programs to be ineffective. Contradictory to our results, reviews by Smith et al. [43] and Meng et al. [44] showed that cardiorespiratory programs were effective for decreasing pain, but they did not find a beneficial effect of core strength training, as compared to other exercise therapy modes, on improving pain. Wewege et al. recently found no superiority of either mode [14]. This high variability in effectiveness of exercise programs, according to the authors, might result from a variety of factors of the included studies, such as low adherence rates, high heterogeneity in patient characteristics, and unclear definitions of the study protocols or therapy modalities, such as training duration and frequency [14,15]. However, while high exercise intensity was noted to be important to improve the outcomes [15,44], none of the previous reviews provided specific information concerning the intensity used in the examined programs, making it difficult to compare the results displayed there to the ones found in our study.

The current study implemented HIT protocols and used clear definitions for each training mode. Furthermore, each group received a therapy with comparable training duration, volume, and frequency, so that these factors could not impact the final outcomes. Results clearly showed that there were no major differences in effectiveness when comparing exercise modes. Moreover, strength outcomes were limited in all groups, as no group achieved minimal detectable change values [37]. Results were contradictory to what the authors expected, as it has been previously displayed that a combination of a HIT cardiorespiratory program with a HIT strength program further improves the outcomes of exercising in healthy persons [27]. Therefore, the authors assumed the HITCOM, HITSTAB, and HITSTRE groups to improve superiorly. The latter groups all combined at least two HIT protocols, while the HITMOB group only performed a HIT cardiorespiratory protocol (as mobility exercises were the only exercises not being able to be executed at high intensity). As the previously known methodological issues were largely resolved in the current study, these outcomes can also support the idea that exercise therapy effectiveness might be due to something other than local effects, such as the enhancement of exercise capacity, strength gains, or the improved core stability [45]. Indeed, recent research has stated that exercise therapy actually might work more through central effects/mechanisms not related to physical performance outcomes [46,47]. These comprise of both psychosocial factors, such as fear-avoidance or self-efficacy beliefs, and neurophysiological factors, such as an altered pain processing or correction of a distorted body scheme. Recent research found that, possibly, the high-intensity cardiorespiratory interval training already provided sufficient centrally mediated stimuli by itself to provide effective therapy results in this population. To provide more detailed statements on the impact of central mechanisms of HIT, more research is needed that is focused on these underlying mechanisms.

The present study has several strengths. Firstly, it was the largest RCT intervention study performed on the subject of HIT exercise therapy in the rehabilitation of persons with NSCLBP. Secondly, groups were volume-adjusted, meaning that the volume of training was the same in each group. This was a necessity to evaluate the results without creating more heterogeneity of therapy modalities as displayed in earlier studies. Thirdly, clear reproducible definitions were provided concerning how HIT can be set up for each training mode in CNSLBP. As no adverse events were noted, and no drop out because of low back pain was described, the authors are certain that these protocols are feasible. As such, this article provides rehabilitation protocols that can be used directly in clinical settings where exercise therapy for CNSLBP might be considered.

The present study also has some limitations. Firstly, while no statistical differences between the groups were found, a post hoc power analysis showed the power for this analysis to be to low (1-β = 0.39), and this might have produced a false-negative outcome. However, the authors argue that the absolute differences between groups were of a small order of magnitude, which would severely limit the possibility of a greater sample to produce clinically relevant differences. Therefore, the authors believe that this would not change the clinical implications of the current results. Secondly, although a group was incorporated that only performed cardiorespiratory training without the addition of a strength protocol (i.e., HITMOB), the isolated effect of high-intensity cardiorespiratory interval training as a standalone therapy could not be fully displayed. Thirdly, this study did not elaborate on the impact of other factors that have been found to be related with the persistence of CNSLBP. As CNSLBP has been displayed as a disorder with a multidimensional cause and with a heterogeneous population [48], results might be dependent of or affected by other outcomes specific to CNSLBP, such as pain related factors (e.g., central sensitization and exercise-induced hyperalgesia) or psychosocial factors (e.g., fear of movement, self-efficacy, and psychological distress). As discussed above, these might have a more decisive effect than the mode of training or are affected by therapy modes that did not show added effect for the currently described outcomes. Further research is needed to evaluate their effect on the intervention outcomes. Fourthly, as the participants of this study were recruited for an exercise therapy study, expectations and beliefs toward this therapy method could have deviated from the average person’s with CNSLBP (i.e., selection bias toward persons who engage in active treatment approaches). Fifthly, no blinding of outcome assessors was performed, due to practical limitations of the research unit. To limit influence of the outcome assessors (i.e., observer bias), assessment instruments were stripped of data displays while assessments were executed, and a strict standardized testing methodology was used. Lastly, this study did not report a long-term follow-up. As such, adherence to the training mode outside of a rehabilitation setting and retention of therapy effects could not be evaluated.

## 5. Conclusions

All four HIT groups in this study displayed considerable clinically relevant improvements. As such, cardiorespiratory high-intensity interval training effectively improves CNSLBP rehabilitation irrespective of the addition of another HIT exercise mode or a mobility protocol. When setting up exercise therapy programs in CNSLBP rehabilitation, this HIT modality can be combined with other frequently used therapy modes; however, no differences in effectiveness should thereby be expected. These outcomes show a promising value of exercise therapy at high-intensity in CNSLBP rehabilitation. More research is needed to evaluate the underlying working mechanisms of HIT and response to exercise therapy of persons with specific characteristics related to the disorder.

## Figures and Tables

**Table 1:** Demographic and clinical characteristics of participants at baseline.

Variables	HITCOM (*n* = 19)	HITSTRE (*n* = 21)	HITSTAB (*n* = 20)	HITMOB (*n* = 20)	*p*-Value
Gender (m/f)	6/13	8/13	8/12	12/8	
Age (years)	44.9 (8.6)	46.4 (9.5)	42.0 (10.9)	42.7 (9.3)	0.162
Symptom duration (years)	14.3 (8.3)	15.0 (8.8)	8.8 (6.0)	15.8 (11.1)	0.001
BMI (kg/m^2^)	25.4 (4.0)	25.4 (4.1)	23.7 (3.4)	25.1 (2.3)	0.232
Physical activity (PASIPD)	15.7 (10.4)	14.2 (10.9)	16.5 (12.7)	17.8 (12.2)	0.891
Kinesiophobia (TSK)	32.0 (6.0)	34.3 (5.4)	33.1 (5.4)	36.6 (6.4)	0.131

Categorical variables are expressed as number (%), and continuous variables are expressed as mean (SD). The *p*-values represent between-group analyses.

**Table 2:** Results of the outcome measures collected from participants at PRE and POST, together with between-group differences.

	HITCOM (*n* = 18)			HITSTRE (*n* = 19)			HITSTAB (*n* = 20)			HITMOB (*n* = 17)			Interaction	
Variables	PRE	POST	Δ	PRE	POST	Δ	PRE	POST	Δ	PRE	POST	Δ	*p*-Value	CI
*Pain intensity*	5.7	2.5	−3.2 *	5.1	3.1	−2.0 *	5.9	2.8	−3.2 *	6.0	2.5	−3.4 *	0.176	(3.9;4.5)
NPRS, 0–10	(1.3)	(1.2)	(1.5)	(1.9)	(1.8)	(1.9)	(1.3)	(2.1)	(2.3)	(1.4)	(1.5)	(2.0)		
*Disability*	22.8	7.8	−14.6 *	20.0	14.6	−5.4 *	22.0	12.4	−9.6 *	21.6	12.2	−9.6 *	0.107	(7.4;9.1)
MODI, %	(9.4)	(5.6)	(8.0)	(10.2)	(10.0)	(10.6)	(11.2)	(4.8)	(12.2)	(9.4)	(8.0)	(7.4)		
*Functioning*	44	70	26 *	42	64	21 *	48	64	18 *	36	66	32 *	0.334	(51;58)
PSFS, %	(17)	(15)	(20)	(17)	(18)	(18)	(18)	(14)	(19)	(16)	(21)	(25)		
*Exercise capacity*	31.2	36.1	4.4 *	33.1	36.3	3.2 *	34.9	37.3	2.4 *	33.3	36.4	3.0 *	0.191	(33.0;36.9)
VO2max, mL/kg/min	(9.3)	(8.0)	(3.5)	(10.6)	(11.1)	(4.2)	(4.7)	(4.5)	(3.4)	(8.4)	(9.4)	(3.6)		
*Exercise capacity*	14.3	17.0	2.5 *	14.6	16.1	1.6 *	14.6	16.8	2.2 *	14.1	16.2	2.2 *	0.193	(14.8;16.2)
Cycling time, min	(3.8)	(3.5)	(1.0)	(3.8)	(3.1)	(1.6)	(2.5)	(2.2)	(1.1)	(2.8)	(3.0)	(1.0)		
*Muscle strength*	1.40	1.45	0.04	1.33	1.28	−0.05	1.49	1.59	0.09	1.42	1.58	0.17 *	0.218	(1.38;1.52)
Abdominal, Nm/kg	(0.29)	(0.28)	(0.18)	(0.36)	(0.44)	(0.53)	(0.27)	(0.25)	(0.19)	(0.43)	(0.43)	(0.12)		
*Muscle strength*	3.10 (0.84)	3.49	0.31 *	3.06	3.16	0.11	3.36	3.44	0.08	3.02	3.06	0.04	0.527	(3.03;3.42)
Back, Nm/kg		(0.73)	(0.45)	(1.10)	(0.91)	(0.59)	(0.70)	(0.70)	(0.47)	(1.14)	(0.92)	(0.45)		

Values in PRE, POST, and delta (Δ) are reported as mean (standard deviation) and represent results of the Numeric Pain Rating Scale (NPRS), Modified Oswestry Disability Index (MODI), Patient Specific Functioning Scale (PSFS), a cardiopulmonary exercise capacity test, and a maximum isometric muscle strength test of the abdominals and back, before (PRE), and after (POST) a 12-week (2x/week, 24 sessions) program of high-intensity cardiorespiratory interval training coupled with either a combined program of high-intensity general resistance and core strength training (HITCOM), high-intensity general resistance training (HITSTRE), high-intensity core strength training (HITSTAB), or a trunk mobility program (HITMOB). Delta displays the post–pre time difference. * Two-sided within group differences *p* < 0.05; † two-sided between group differences *p* < 0.05. CI shows the 95% confidence intervals of the mixed-model results.
